# Immune checkpoint inhibitor-associated eosinophilic fasciitis: a systematic review of reported cases^؆^

**DOI:** 10.1016/j.abd.2026.501422

**Published:** 2026-08-10

**Authors:** Yahya Argobi, Faisal Tobeigei, Faris I. Alasiri

**Affiliations:** a Department of Dermatology, College of Medicine, King Khalid University, Abha, Saudi Arabia; b Security Forces Hospitals Program, General Directorate of Medical Services, Ministry of Interior, Riyadh, Saudi Arabia

**Keywords:** Eosinophilia, Fasciitis, Immune checkpoint inhibitors, Immunotherapy, Neoplasms

## Abstract

**Background:**

Eosinophilic Fasciitis (EF) is a rare immune-mediated fibrosing disorder of the fascia. Immune Checkpoint Inhibitors (ICIs) have transformed oncologic therapy but may precipitate immune-related Adverse Events (irAEs), including dermatologic and rheumatologic manifestations. ICI-associated EF has been increasingly reported, yet remains poorly characterized.

**Objective:**

To systematically summarize the clinical features, diagnostic approaches, management strategies, and outcomes of ICI-associated EF.

**Methods:**

A systematic review was conducted in accordance with PRISMA 2020. PubMed/MEDLINE, EMBASE, and Web of Science were searched from inception through January 2026. Eligible studies were case reports, case series, or observational studies describing adult cancer patients who developed EF temporally associated with ICI therapy. Two reviewers independently screened studies, extracted data, and assessed methodological quality using Joanna Briggs Institute tools.

**Results:**

Among 148 records identified, 93 remained after removal of 55 duplicates; 28 full-text articles were assessed and 18 met inclusion criteria, representing 22 unique patients. Nivolumab and pembrolizumab were the most frequently implicated agents. EF onset ranged from several weeks to months after ICI initiation. The most commonly affected regions were the extremities. Diagnosis was confirmed by histopathology and/or imaging in most cases. Systemic corticosteroids were the main treatment, with additional immunosuppressive agents used in selected patients. Most cases showed partial or complete clinical improvement.

**Study limitations:**

Evidence is limited to case reports and small case series, with heterogeneous reporting, precluding incidence estimation and limiting generalizability.

**Conclusions:**

ICI-associated EF is a rare but clinically relevant irAE with dermatologic and connective tissue involvement. Early recognition and multidisciplinary management are essential to prevent long-term morbidity.

## Introduction

Immune checkpoint inhibitors (ICIs) have revolutionized the treatment of multiple malignancies by enhancing antitumor T-cell responses through blockade of immune regulatory pathways, including PD-1, PD-L1, and CTLA-4.^1^, ^2^, ^3^ These agents improve survival outcomes but may disrupt immune tolerance and induce immune-related Adverse Events (irAEs) affecting multiple organ systems.^1^, ^4^, ^5^

Cutaneous, musculoskeletal, and connective tissue irAEs are increasingly recognized in routine practice.^5^ Among these, eosinophilic fasciitis (EF) is a rare fibrosing disorder with prominent cutaneous and fascial manifestations, including skin induration and limb stiffness.^5^, ^6^, ^7^, ^8^ First described by Shulman in 1974, EF is characterized by fascial inflammation, progressive fibrosis, peripheral eosinophilia, and peau d’orange-like changes, with diagnosis typically relying on full-thickness biopsy and/or characteristic Magnetic Resonance Imaging (MRI) findings.^6^, ^7^, ^8^

Although individual cases of ICI-associated EF have been reported, its overall clinical spectrum, diagnostic pathways, management strategies, and outcomes remain incompletely defined.^9^, ^10^, ^11^, ^12^, ^13^, ^14^, ^15^, ^16^, ^17^, ^18^, ^19^, ^20^, ^21^, ^22^, ^23^, ^24^, ^25^, ^26^ The authors therefore conducted a systematic review to synthesize current evidence regarding ICI-associated EF, with a focus on features relevant to dermatology and onco-dermatology practice.

## Methods

### Study design and registration

This systematic review was conducted in accordance with the Preferred Reporting Items for Systematic Reviews and Meta-Analyses (PRISMA 2020) guidelines.^27^ The protocol was prospectively registered in PROSPERO (CRD420251134379). Because only previously published data were analyzed, institutional review board approval was not required.

### Review framework

The review question was structured using the population-exposure-outcome (PEO) framework: Population, adult patients with malignancy; Exposure, immune checkpoint inhibitors (PD-1, PD-L1, or CTLA-4 inhibitors); Outcome, development of eosinophilic fasciitis.

### Search strategy

A comprehensive search was performed in PubMed/MEDLINE (Ovid), EMBASE (Ovid), and Web of Science from database inception through January 2026. Search terms combined controlled vocabulary (MeSH/Emtree) and free-text keywords related to immune checkpoint inhibitors and eosinophilic fasciitis, including “Shulman syndrome.” The full search strategies for each database are provided in Supplementary Appendix A.

### Eligibility criteria

Studies were eligible if they met all of the following criteria: 1) Case report, case series, or observational study design; 2) Adult oncology patients exposed to ICIs; 3) Eosinophilic fasciitis temporally associated with ICI therapy; and 4) Full-text article available in English.

Exclusion criteria were: 1) Reviews, editorials, or conference abstracts without extractable patient-level data; 2) Pharmacovigilance database analyses without clinical confirmation; 3) EF-like or scleroderma-like cases without confirmed diagnosis; 4) Non-ICI-related EF; and 5) Animal or laboratory studies.

A detailed list of excluded full-text articles and reasons for exclusion is provided in Supplementary Table S1.

### Study selection

After removal of duplicates, two reviewers independently screened titles and abstracts. Potentially eligible articles were retrieved in full text and assessed against the inclusion and exclusion criteria. Discrepancies were resolved by discussion and consensus.

### Data extraction

Two reviewers independently extracted data using a standardized form. Extracted variables included patient demographics, underlying malignancy, ICI agent and regimen, time from ICI initiation to EF onset, anatomical regions affected, diagnostic modality (biopsy and/or imaging), treatment strategies, and clinical outcomes.

### Quality assessment

Methodological quality of the included case reports and case series was evaluated using the appropriate Joanna Briggs Institute (JBI) critical appraisal checklists.^28^ Two reviewers applied the tools independently, and disagreements were resolved by consensus.

### Data synthesis

Given the rarity of ICI-associated EF and the absence of comparable cohort data, a quantitative meta-analysis was not feasible. Findings were synthesized narratively, with a descriptive summary of study- and patient-level characteristics.

## Results

### Study selection

**Figure 1:**
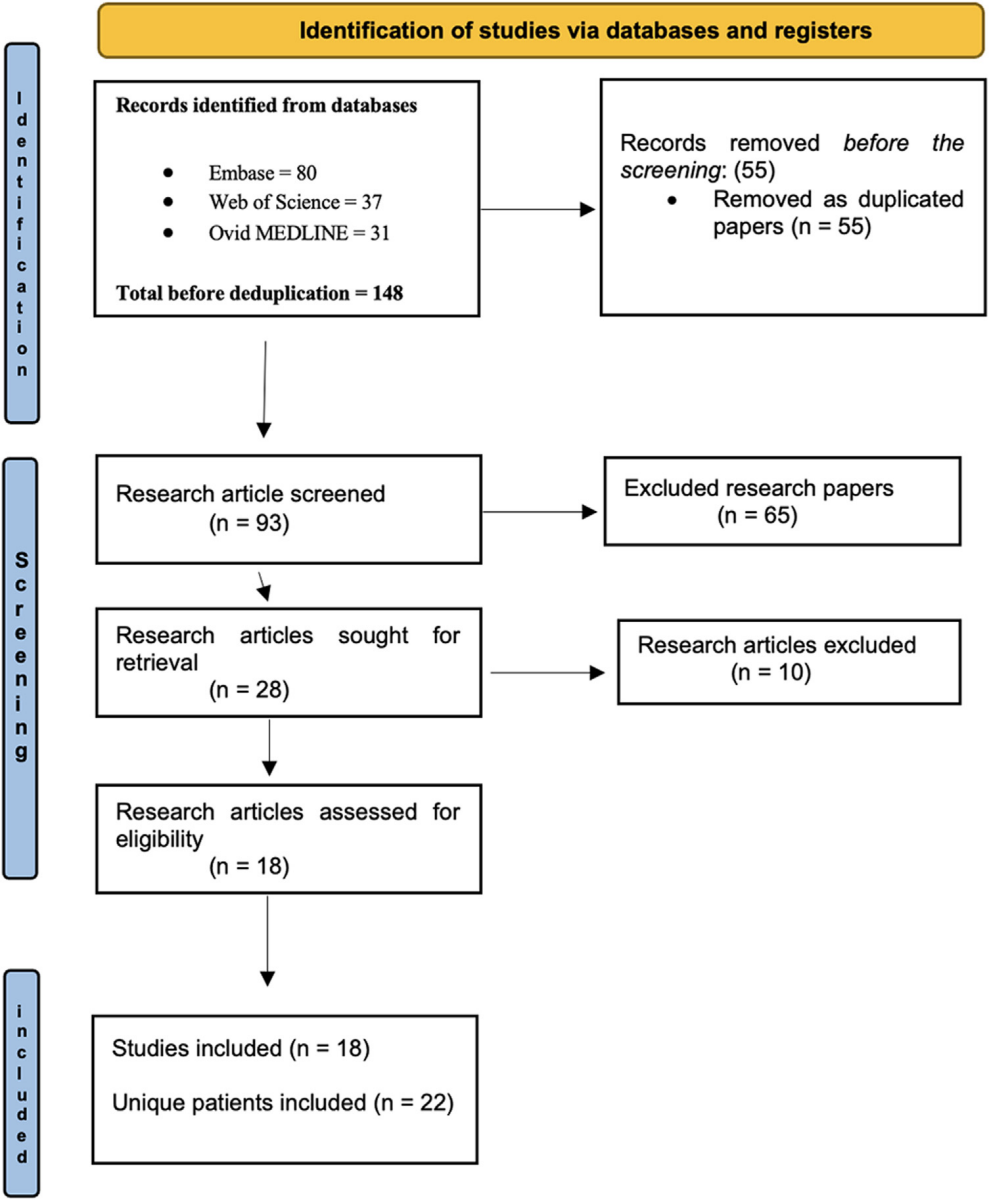
PRISMA Flowchart for screening and selection of included studies.

A total of 148 records were identified (EMBASE: 80; MEDLINE: 31; Web of Science: 37). After removal of 55 duplicates, 93 records remained for title and abstract screening, of which 65 were excluded. Twenty-eight full-text articles were assessed, and 10 were excluded for predefined reasons (Supplementary Table S1). Eighteen studies met the inclusion criteria,^9^, ^10^, ^11^, ^12^, ^13^, ^14^, ^15^, ^16^, ^17^, ^18^, ^19^, ^20^, ^21^, ^22^, ^23^, ^24^, ^25^, ^26^ representing 22 unique patients. The study selection process is summarized in Fig. 1.

### Study characteristics

All included studies were individual case reports or small case series published between 2016 and 2025, originating from North America and Europe, and involved a range of solid tumors treated with ICIs.

The most frequently implicated immune checkpoint inhibitors were nivolumab and pembrolizumab, with occasional use of combination therapy or alternative agents.

Detailed characteristics of the included studies are presented in Table 1.^9^, ^10^, ^11^, ^12^, ^13^, ^14^, ^15^, ^16^, ^17^, ^18^, ^19^, ^20^, ^21^, ^22^, ^23^, ^24^, ^25^, ^26^

**Table 1:** Characteristics of included studies reporting ICI-associated eosinophilic fasciitis

First Author	Year	Country	Study Type	ICI Agent(s)	Number of EF Cases
Khoja	2016	Canada	Case report	Pembrolizumab	1
Andrés-Lencina	2018	Spain	Case report	Nivolumab ± Ipilimumab	1
Le Tallec	2019	France	Case report	Nivolumab	1
Toussaint	2019	Germany	Case report	Ipilimumab → Pembrolizumab	1
Wissam	2019	Belgium	Case report	Atezolizumab	1
Chan	2020	USA	Case series	Atezolizumab / Nivolumab / Pembrolizumab	4
Bui	2020	USA	Case report	Nivolumab	1
Ollier	2020	France	Case report	Nivolumab	1
Pabón-Cartagena	2020	USA	Case report	Nivolumab	1
Lacombe	2021	France	Case report	Nivolumab	1
Bourcier	2021	Canada	Case report	Pembrolizumab	1
Boppana	2021	USA	Case report	Cemiplimab	1
Zampeli	2021	Greece	Case report	Pembrolizumab	1
Haroon	2022	USA	Case report	Ipilimumab + Nivolumab	1
Amrane	2022	France	Case report	Pembrolizumab	1
Benzaquen	2023	France	Case report	Nivolumab	1
Oke	2025	USA	Case report	Pembrolizumab	1
Biteau	2025	France	Case series	Nivolumab / Pembrolizumab	3

Total studies: 18.

Total unique patients: 22.

### Patient characteristics and clinical presentation

Among the 22 patients, melanoma and non-small cell lung cancer were the most common underlying malignancies. Nivolumab and pembrolizumab were the most frequently implicated ICIs, followed by other PD-1/PD-L1 or CTLA-4 inhibitors. The extremities were the most frequently affected anatomical regions. Time from ICI initiation to EF onset ranged from several weeks to many months. Clinical manifestations typically included progressive skin induration, limb edema, decreased range of motion, and peripheral eosinophilia. Diagnosis was confirmed in most cases by full-thickness biopsy and/or characteristic MRI findings. Detailed patient-level clinical characteristics are summarized in Table 2.^9^, ^10^, ^11^, ^12^, ^13^, ^14^, ^15^, ^16^, ^17^, ^18^, ^19^, ^20^, ^21^, ^22^, ^23^, ^24^, ^25^, ^26^

**Table 2:** Patient-level clinical features of included cases

First Author	Age	Sex	Cancer Type	ICI	Time to EF	Region Affected	Eosinophilia	Diagnosis Method
Khoja	NR	M	Melanoma	Pembrolizumab	Months	Upper limbs	Yes	Biopsy
Andrés-Lencina	NR	NR	Melanoma	Nivolumab	Months	Limbs	Yes	Biopsy
Le Tallec	NR	NR	Lung cancer	Nivolumab	Months	Limbs	Yes	Biopsy
Toussaint	NR	NR	Melanoma	Ipilimumab → Pembrolizumab	Months	Limbs	Yes	Biopsy
Wissam	NR	F	Breast cancer	Atezolizumab	Months	Limbs	Yes	Biopsy + MRI
Chan (4 cases)	NR	NR	Mixed	Mixed	NR	Limbs	Yes	Biopsy
Bui	NR	NR	Melanoma	Nivolumab	Months	Limbs	Yes	Biopsy
Ollier	NR	M	Melanoma	Nivolumab	Months	Limbs	Yes	Biopsy
Pabón-Cartagena	NR	F	Cancer	Nivolumab	Months	Limbs	Yes	Biopsy
Lacombe	NR	NR	Melanoma	Nivolumab	Months	Limbs	Yes	Biopsy
Bourcier	NR	NR	Melanoma	Pembrolizumab	Months	Limbs	Yes	Biopsy
Boppana	NR	NR	Skin cancer	Cemiplimab	Months	Limbs	Yes	Biopsy
Zampeli	NR	NR	Cancer	Pembrolizumab	Months	Limbs	Yes	Biopsy
Haroon	NR	NR	Cancer	Ipi + Nivo	Months	Limbs	Yes	Biopsy
Amrane	NR	NR	Cancer	Pembrolizumab	Months	Limbs	Yes	MRI + Biopsy
Benzaquen	NR	NR	Cancer	Nivolumab	Months	Limbs	Yes	Biopsy
Oke	NR	NR	Bladder cancer	Pembrolizumab	Months	Limbs	Yes	Biopsy
Biteau (3 cases)	NR	NR	Mixed	Mixed	Weeks-Months	Limbs	Yes	Biopsy

### Management and outcomes

Systemic corticosteroids were the primary treatment in the majority of patients. Additional immunosuppressive agents, most commonly methotrexate or mycophenolate mofetil, and less frequently sirolimus, intravenous immunoglobulin, or biologic therapies, were used in steroid-refractory or relapsing cases. Discontinuation of immune checkpoint inhibitor therapy was reported in many cases, although some patients continued treatment without interruption. Most patients achieved partial or complete clinical improvement, and no deaths were directly attributed to EF. Detailed treatment strategies and outcomes are presented in Table 3.^9^, ^10^, ^11^, ^12^, ^13^, ^14^, ^15^, ^16^, ^17^, ^18^, ^19^, ^20^, ^21^, ^22^, ^23^, ^24^, ^25^, ^26^

**Table 3:** Patient-level treatment and outcomes

First Author	Steroids	Second-line Therapy	ICI Stopped	Outcome
Khoja	Yes	No	Yes	Improved
Andrés-Lencina	Yes	No	Yes	Improved
Le Tallec	Yes	Sirolimus	Yes	Improved
Toussaint	Yes	No	Yes	Improved
Wissam	No	No	Yes	Improved
Chan	Yes	MTX/MMF	NR	Improved
Bui	No	No	No	Improved
Ollier	Yes	No	Yes	Improved
Pabón-Cartagena	Yes	No	Yes	Improved
Lacombe	Yes	No	Yes	Improved
Bourcier	No	No	No	Improved
Boppana	Yes	No	Yes	Improved
Zampeli	Yes	No	Yes	Improved
Haroon	Yes	No	Yes	Improved
Amrane	Yes	No	Yes	Improved
Benzaquen	Yes	No	Yes	Improved
Oke	Yes	No	Yes	Improved
Biteau	Yes	MTX	NR	Improved

### Quality assessment

The methodological quality of the included studies was generally high based on the Joanna Briggs Institute critical appraisal criteria. Most studies provided clear patient descriptions, diagnostic confirmation, and outcome reporting.

A detailed assessment of methodological quality for each included study is presented in Table 4.^9^, ^10^, ^11^, ^12^, ^13^, ^14^, ^15^, ^16^, ^17^, ^18^, ^19^, ^20^, ^21^, ^22^, ^23^, ^24^, ^25^, ^26^

**Table 4:** Methodological quality assessment (JBI Checklist)

Study	Clear Patient Description	History Timeline	Diagnostic Methods	Intervention Described	Post-intervention Outcome	Adverse Events	Takeaway Lessons	Overall
Khoja	Yes	Yes	Yes	Yes	Yes	No	Yes	High
Andrés-Lencina	Yes	Yes	Yes	Yes	Yes	No	Yes	High
Le Tallec	Yes	Yes	Yes	Yes	Yes	No	Yes	High
Toussaint	Yes	Yes	Yes	Yes	Yes	No	Yes	High
Wissam	Yes	Yes	Yes	Yes	Yes	No	Yes	High
Chan	Yes	Yes	Yes	Yes	Yes	No	Yes	High
Bui	Yes	Yes	Yes	Yes	Yes	No	Yes	High
Ollier	Yes	Yes	Yes	Yes	Yes	No	Yes	High
Pabón-Cartagena	Yes	Yes	Yes	Yes	Yes	No	Yes	High
Lacombe	Yes	Yes	Yes	Yes	Yes	No	Yes	High
Bourcier	Yes	Yes	Yes	Yes	Yes	No	Yes	High
Boppana	Yes	Yes	Yes	Yes	Yes	No	Yes	High
Zampeli	Yes	Yes	Yes	Yes	Yes	No	Yes	High
Haroon	Yes	Yes	Yes	Yes	Yes	No	Yes	High
Amrane	Yes	Yes	Yes	Yes	Yes	No	Yes	High
Benzaquen	Yes	Yes	Yes	Yes	Yes	No	Yes	High
Oke	Yes	Yes	Yes	Yes	Yes	No	Yes	High
Biteau	Yes	Yes	Yes	Yes	Yes	No	Yes	High

## Discussion

This systematic review identified 22 unique cases of eosinophilic fasciitis temporally associated with immune checkpoint inhibitor therapy, confirming that this is a rare but clinically relevant irAE with both dermatologic and connective tissue involvement. Nivolumab and pembrolizumab were the most frequently implicated agents, likely reflecting both their widespread use and accumulated post-marketing experience.^9^, ^10^, ^11^, ^12^, ^13^, ^14^, ^15^, ^16^, ^17^, ^18^, ^19^, ^20^, ^21^, ^22^, ^23^, ^24^, ^25^, ^26^ Consistent with the patient-level data (Table 2), the extremities were the most frequently affected anatomical regions.

Compared with broader pharmacovigilance and multicenter cohorts, our strictly case-based approach captured fewer patients. For example, Biteau et al. reported three new cases within a larger French series of ICI-related EF, but many pharmacovigilance-derived or EF-like signals did not meet our requirement for confirmed EF and extractable patient-level data. ^26^ This stringent case definition, while restrictive, enhances diagnostic certainty and clinical interpretability. A detailed methodological appraisal of included studies is presented in Table 4.

The largest single case aggregation among the included reports was the series by Chan et al., which described four patients with biopsy-confirmed EF following checkpoint inhibitor therapy, underscoring the rarity of this complication even in high-volume centers.^14^ The available data suggest that ICI-associated EF shares pathophysiologic features with idiopathic EF, including immune dysregulation with eosinophilic infiltration and fibroblast activation.^4^, ^5^, ^6^ Management in published cases has largely mirrored that of conventional EF, with systemic corticosteroids as first-line therapy and escalation to steroid-sparing agents in refractory or relapsing disease.^4^, ^5^, ^6^, ^7^, ^8^ As detailed in Table 3, most patients demonstrated clinical improvement following corticosteroid therapy, with additional immunosuppressive agents required in selected cases.

Despite generally favorable outcomes, delayed recognition may permit progression to fixed fibrosis and persistent functional impairment. The presence of new or worsening skin induration, limb edema, or restricted mobility in patients receiving ICIs should therefore prompt early dermatologic and rheumatologic evaluation, with consideration of EF in the differential diagnosis. These findings highlight the importance of integrating dermatologic assessment into the multidisciplinary evaluation of patients receiving immune checkpoint inhibitors.

### Limitations

This review is limited by its reliance on case reports and small case series, which are prone to selection and publication bias and often lack standardized reporting. Heterogeneity in diagnostic criteria, imaging, and histopathologic evaluation, and treatment strategies further restricts comparability across cases. As a result, neither incidence nor definitive risk factors for ICI-associated eosinophilic fasciitis can be determined from the available data.

## Conclusion

Immune checkpoint inhibitor–associated eosinophilic fasciitis is an uncommon but clinically important immune-related adverse event with both dermatologic and connective tissue involvement that should be recognized by dermatologists, oncologists, and rheumatologists. Early diagnosis and coordinated multidisciplinary management, with timely initiation of immunosuppressive therapy when indicated, appear crucial to limiting long-term functional impairment. Larger prospective and multicenter studies are needed to clarify predisposing factors, refine diagnostic pathways, and optimize therapeutic strategies.

## ORCID IDs

Faisal Tobeigei: 0000-0003-3906-2017

Faris I. Alasiri: 0009-0009-0149-6531

## Prospero registration

CRD420251134379.

## Research data availability

The entire dataset supporting the results of this study was published in this article.

## Financial support

This work was supported by King Khalid University's Deanship of Scientific Research [grant numbers GRP/122/44].

## Authors' contributions

Yahya Argobi: Approval of the final version of the manuscript; critical literature review; data collection, analysis and interpretation; effective participation in research orientation; intellectual participation in propaedeutic and/or therapeutic management of studied cases; manuscript critical review; preparation and writing of the manuscript; statistical analysis; study conception and planning.

Faisal Tobeigei: Approval of the final version of the manuscript; critical literature review; data collection, analysis and interpretation; effective participation in research orientation; intellectual participation in propaedeutic and/or therapeutic management of studied cases; manuscript critical review; preparation and writing of the manuscript; statistical analysis; study conception and planning.

Faris I. Alasiri: Approval of the final version of the manuscript; critical literature review; data collection, analysis and interpretation; effective participation in research orientation; intellectual participation in propaedeutic and/or therapeutic management of studied cases; manuscript critical review; preparation and writing of the manuscript; statistical analysis; study conception and planning.

## Conflicts of interest

None declared.
