# Supplementary material for: Immune checkpoint inhibitor-associated eosinophilic fasciitis: a systematic review of reported cases
Source: An Bras Dermatol. 2026 Aug 10;101(5):501422. doi: 10.1016/j.abd.2026.501422 (PMC13485498; doi:10.1016/j.abd.2026.501422)
Supplement: Supplementary file 1 [file mmc1.docx]

ABD-D-25-00932_Supplementary material

**Supplementary Table S1** Full-text articles excluded after eligibility assessment (n=10).

| **First Author** | **Year** | **Journal / Source** | **Reason for Exclusion (PRISMA-compatible)** |
| --- | --- | --- | --- |
| Ashraf | 2023 | *Br J Dermatol*(Supplement/Abstract) | Conference abstract; insufficient patient-level extractable data |
| Huang | 2022 | *Ann Rheum Dis* (EULAR abstract) | Conference abstract; no extractable patient-level data |
| Le Tallec | 2020 | *J Thorac Oncol* | Duplicate patient: follow-up/therapeutic update of previously published 2019 case |
| Salamaliki | 2020 | *Rheumatology and Therapy* | EF-like / Scleroderma-like syndrome (mimicker); not confirmed eosinophilic fasciitis case definition |
| Lyu | 2025 | *Int J Cancer* | Pharmacovigilance (FAERS) analysis; no patient-level clinical confirmation/extraction |
| Ganta | 2022 | *Cureus* | Not EF outcome (reports other irAEs, not eosinophilic fasciitis) |
| Abdel-Wahab | 2019 | *Rheumatology*(Supplement) | Review/overview; not original EF cases with extractable patient-level data |
| Joly-Chevrier | 2023 | *Cancers* | Scoping review; not original patient-level EF cases |
| Knapp | 2024 | *Rev Med Interne* | Narrative review (pathophysiology/therapeutics); not original ICI-EF cases |
| Teboul | 2022 | *J Am Acad Dermatol* | Pharmacovigilance analysis (drug-induced EF); not ICI-specific patient-level EF cases and not extractable cases |

**Supplementary Appendix 1**

Detailed Search Strategies

**Supplementary Appendix 1** Full search strategies for systematic review.

| **Search Date** | |
| --- | --- |
| All databases were searched from inception through **January 2026**. | |
|  | |
| **1. EMBASE (Ovid)** | |
| **Platform:** Ovid EMBASE | |
| **Records retrieved:** 80 | |
| 1. exp immune checkpoint inhibitor/ | |
| 2. (checkpoint inhibitor* or immune checkpoint inhibitor*).tw,kf. | |
| 3. nivolumab.tw,kf. | |
| 4. pembrolizumab.tw,kf. | |
| 5. ipilimumab.tw,kf. | |
| 6. atezolizumab.tw,kf. | |
| 7. durvalumab.tw,kf. | |
| 8. avelumab.tw,kf. | |
| 9. cemiplimab.tw,kf. | |
| 10. 1 or 2 or 3 or 4 or 5 or 6 or 7 or 8 or 9 | |
| 11. exp eosinophilic fasciitis/ | |
| 12. (eosinophilic fasciitis or Shulman syndrome).tw,kf. | |
| 13. 11 or 12 | |
| 14. 10 and 13 | |
| Limits applied: | |
| • Humans | |
| • No language restriction at search stage | |
|  | |
| **2. MEDLINE (Ovid)** | |
| **Platform:** Ovid MEDLINE(R) All | |
| **Records retrieved:** 31 | |
| 1. exp Immune Checkpoint Inhibitors/ | |
| 2. (checkpoint inhibitor* or immune checkpoint inhibitor*).tw. | |
| 3. nivolumab.tw. | |
| 4. pembrolizumab.tw. | |
| 5. ipilimumab.tw. | |
| 6. atezolizumab.tw. | |
| 7. durvalumab.tw. | |
| 8. avelumab.tw. | |
| 9. cemiplimab.tw. | |
| 10. 1 or 2 or 3 or 4 or 5 or 6 or 7 or 8 or 9 | |
| 11. exp Fasciitis/ | |
| 12. (eosinophilic fasciitis or Shulman syndrome).tw. | |
| 13. 11 or 12 | |
| 14. 10 and 13 | |
| Limits applied: | |
| • Humans | |
|  | |
| **3. Web of Science Core Collection** | |
| **Records retrieved:** 37 | |
| Search conducted in Topic field (TS): | |
| TS = ( | |
| ("immune checkpoint inhibitor*" OR nivolumab OR pembrolizumab OR ipilimumab | |
| OR atezolizumab OR durvalumab OR avelumab OR cemiplimab) | |
| AND | |
| ("eosinophilic fasciitis" OR "Shulman syndrome") | |
|  | |
| Indexes searched: | |
| • SCI-Expanded | |
| • ESCI | |
| • Conference Proceedings Citation Index | |
|  | |
| **Combined Results** | |
| **Database** | **Records Retrieved** |
| EMBASE | 80 |
| MEDLINE | 31 |
| Web of Science | 37 |
| Total | 148 |

Duplicates removed: 55. Records screened: 93.
